# Measuring Accelerated Rates of Insertions and Deletions Independent of Rates of Nucleotide Substitution

**DOI:** 10.1007/s00239-016-9761-9

**Published:** 2016-10-21

**Authors:** Omar Navarro Leija, Sanju Varghese, Mira V. Han

**Affiliations:** 1 School of Life Sciences, University of Nevada, Las Vegas, NV 89154 USA; 2 Department of Computer Science, University of Nevada, Las Vegas, NV 89154 USA; 3 Nevada Institute of Personalized Medicine, Las Vegas, NV 89154 USA

**Keywords:** Insertions, Deletions, Substitution rate, Evolutionary constraint

## Abstract

Evolutionary constraint for insertions and deletions (indels) is not necessarily equal to constraint for nucleotide substitutions for any given region of a genome. Knowing the variation in indel-specific evolutionary rates across the sequence will aid our understanding of evolutionary constraints on indels, and help us infer how indels have contributed to the evolution of the sequence. However, unlike for nucleotide substitutions, there has been no phylogenetic method that can statistically infer significantly different rates of indels across the sequence space independent of substitution rates. Here, we have developed a software that will find sites with accelerated evolutionary rates specific to indels, by introducing a scaling parameter that only applies to the indel rates and not to the nucleotide substitution rates. Using the software, we show that we can find regions of accelerated rates of indels in the protein alignments of primate genomes. We also confirm that the sites that have high rates of indels are different from the sites that have high rates of nucleotide substitutions within the protein sequences. By identifying regions with accelerated rates of indels independent of nucleotide substitutions, we will be able to better understand the impact of indel mutations on protein sequence evolution.

**Electronic supplementary material:**

The online version of this article (doi:10.1007/s00239-016-9761-9) contains supplementary material, which is available to authorized users.

## Introduction

Different types of mutations are under different mutation and selection dynamics. In terms of protein structure, there can be regions of the protein that are unconstrained for the amino acid compositions, but constrained for their overall length (Grishin 2001; Sandhya et al. 2009; Zhang et al. 2010). Also there can be regulatory regions of the genomes that have requirements in the distance between functional elements for optimal binding (Sætrom et al. 2007; Liu and Posakony 2012). Thus, it is plausible to assume that evolutionary constraint on nucleotide substitutions will be different from the constraint on insertions or deletions for a given region of the genome.

With nucleotide substitutions, we can identify sites in the genome, which are unusually conserved, indicative of evolutionary constraint, or sites that are changing at an unusually fast rate. Several methods are available that utilize the comparative data from multiple species and take into account the phylogeny to estimate different rates across the genome assuming well-developed models of sequence evolution (Pond et al. 2005; Yang 2007; Hubisz et al. 2011). The variation in the evolutionary rates of nucleotide substitutions has been utilized to infer functional elements in the genome, or predict the deleterious effect of point mutations (Ng and Henikoff 2001). Significant progress has been made in the modeling of indel events during sequence evolution (Thorne et al. 1991; McGuire et al. 2001; Redelings and Suchard 2005; Rivas and Eddy 2008), and the indel models have been incorporated into sequence alignments (Redelings and Suchard 2005) and phylogeny inference (Rivas and Eddy 2008). But, unlike for nucleotide substitutions, there is no method that can measure different rates of indels across the sequence space that also utilizes the recently developed phylogenetic indel models. Thus, in order to predict the deleterious effect of indels, instead of directly relying on the evolutionary rates of indel mutations, softwares have used ad hoc aggregation of the nucleotide substitution rates spanning the region of the indel event (Hu and Ng 2012; Zhao et al. 2013). Here, we present a software that combines the indel model by Rivas and Eddy (2008) with the software PHAST (Hubisz et al. 2011) to infer accelerated rates of indels independent of accelerated rates of nucleotide substitutions. The software measures the global rate of insertions and deletions across a set of multiple sequence alignments and identifies sites that are under indel rates different from the estimated global rate. This allows the users to identify regions of the proteins that have experienced unusually high indel rates but not high nucleotide substitution rates, and vice versa. The software could have broad utility in understanding the impact of indels on protein structures, and predicting the effect of indel mutations within a protein.

## Materials and Methods

### Model

Our software is based on the source code of the software package PHAST (Phylogenetic Analysis with Space/Time Models) (Hubisz et al. 2011). The default behavior of PHAST is to consider gaps in the alignment as missing data, although PHAST also has an option (-G) to consider it as a fifth character. Our program allows the estimation of three new models that are not implemented in the original PHAST. The first model is *F84* by Felsenstein and Churchill (Felsenstein and Churchill 1996), the second model is a relaxed version of the indel model *F84ε* as described in Rivas and Eddy (Rivas and Eddy 2008), and the third model is the relaxed *F84ε* plus an extra scale parameter (*ρ*_indel_) that only scales the insertion (*λ*) and deletion (*μ*) rate parameters (Table 1).

**Table 1:** Models newly implemented in the software

Model	*F84* (Felsenstein and Churchill 1996)	*F84ε*-*relaxed*	*F84ε*-*relaxed* + *ρ*_indel_
Parameters	*α*, *β*	*α*, *β*, *λ*, *μ*	*α*, *β*, *λ*, *μ*, *ρ*_indel_

Three models are newly implemented in the extended version of PHAST. The *F84* by Felsenstein and Churchill, *F84ε*-*relaxed* which is a modified version of the model *F84ε* (Rivas and Eddy 2008), and F84*ε*-relaxed + *ρ*_indel_ in which we introduce the scaling parameter to modify the indel rates

*F84ε* (Rivas and Eddy 2008) is a generative model that allows evolutionary histories unconditional on any fixed sequence length. The model involves an extended pruning algorithm that includes four modifications to take into account of the insertion and deletion events: extra bookkeeping in the Felsenstein recursions to enforce that no more than one insertion occurs per column; including a term from the prior ancestral sequence length distribution in the calculation of each individual column-likelihood; including in the overall alignment likelihood the extra normalization terms collected in the “extra column,” denoted * in Rivas and Eddy (2008); and marginalizing the contributions of possible ancestral residues that have left no trace in extant sequences. The extra bookkeeping referred to above is based on the assumption of this model that no more than one insertion occurs per column, in order to ensure all aligned residues are homologous. Although this assumption is valid and important when inferring phylogenies, it does not work for our purpose. Because we rely on multiple recurrent events hitting a single site in order to estimate accelerated rates on a specific region of the genome, we need to allow the clumping of non-homologous indel events into a single column. We explain in more detail, the consequences of this assumption for our problem in the results and in the supplementary materials. To modify the model so it is appropriate for our purpose, we relaxed the assumption to allow multiple insertion events per site (*F84ε*-*relaxed*). This requires removing the extra bookkeeping in the generative model.

So, instead of Eq. (19) in Rivas and Eddy (2008), we allow gaps at the leaves.

1 $$P_{u} \left( {L_{k} , - } \right) = \left\{ {\begin{array}{*{20}l} 1 \hfill & {{\text{if}}\;{\text{leaf}}\;k\;{\text{has }}{-}\;{\text{at }}\;{\text{position}}\;u} \hfill \\ 0 \hfill & {\text{otherwise }} \hfill \\ \end{array} } \right.$$

Instead of Eqs. (20) and (21) in Rivas and Eddy (2008), we just require the general pruning step for any inner nodes.

2 $$P_{u} \left( {L_{k} , i} \right) = \left[ {\left( {\mathop \sum \limits_{1 \le q \le K} P_{u} (L_{{d_{k}^{l} }} ,q)P(q|i,t_{k}^{l} )} \right) + P_{u} (L_{{d_{k}^{l} }} , - )P( - |i,t_{k}^{l} )} \right] \times \left[ {\left( {\mathop \sum \limits_{1 \le s \le K} P_{u} (L_{{d_{k}^{r} }} ,s)P(s|i,t_{k}^{r} )} \right) + P_{u} (L_{{d_{k}^{r} }} , - )P( - |i,t_{k}^{r} )} \right],$$

here *i* includes all possible residues as well as gaps.

Thus, in addition to Eqs. (22), (23), and (24) in Rivas and Eddy (2008), we need to define $P\left( { - |- , t} \right)$. Based on the constraint that the rows of the conditional matrix have to sum to one,

3 $$P\left( { - | - , t} \right) = 1 - \xi_{t}$$

We implemented this relaxed version of the *F84ε* model, called *F84ε*-*relaxed*, in the PHAST software, including the three modifications except for the extra bookkeeping. The program dnaml-ε by Rivas and Eddy also does several extra steps when calculating the likelihood that is different from PHAST. For example, it divides the branch lengths by a constant calculated from the background frequencies, and it performs a midpoint rerooting of the tree. To verify our program was correct we had to get exact results for the likelihood calculations as dnaml-ε. Thus, several options were added to perform the same procedures that dnaml-ε uses. With these options turned on, we confirmed that dnaml-ε and our program computes the same values. In contrast, *F84* does not use a special pruning algorithm, and can be implemented using the native pruning algorithm of PHAST. We also confirmed that when presented with alignments without any gaps, *F84ε* produces the same result as *F84*.

### Estimating the Models

We extended the program phyloFit (Siepel and Haussler 2004) that is part of the software package PHAST to allow the estimation of *F84, F84ε,* and *F84ε*-*relaxed*. Since we were mostly interested in estimating indel rates on codon sequences of the protein-coding regions instead of the whole genome, we modified the software so that it can estimate the model on data consisting of many multiple alignments of different number of sequences instead of one whole-genome alignment.

### Looking for Conservation or Acceleration of Indel Rates Using Likelihood Ratio Test

We extended the program phyloP (Pollard et al. 2010) within PHAST to report new likelihood ratio tests (LRT) for two different model comparisons (Table 2). (1) The base model *F84* is compared with the branch length-scaled model provided by the native function in PHAST. (2) The indel model *F84ε*-*relaxed* is compared against the model *F84ε*-*relaxed* + *ρ*_indel_ which allows a scaling of the insertion deletion rates (*λ* and *μ*) for each site. The latter comparison will identify the sites in the alignment that have significantly higher or significantly lower indel rates than the rates estimated on the whole data.

**Table 2:** Model comparisons newly implemented in the software

Comparison	*d.f.*	Description	Score
*F84* versus *F84* + *ρ*	1	Does scaling on the substitution rates (=scaling branch lengths) improve the model fit?	phyloP
*F84ε*-*relaxed* versus *F84ε*-*relaxed* + *ρ*_indel_	1	Does scaling on the indel rates improve the model fit?	indelP

Two model comparisons are implemented with the likelihood ratio test. First comparison compares *F84* to *F84* + *ρ* using the native branch scaling in the original program phyloP. Second comparison compares *F84ε*-*relaxed* to *F84ε*-*relaxed* + *ρ*_indel_ using our newly implemented indel rate scaling. The *p*-value from the second likelihood ratio test is transformed into an indelP score

In the original PHAST algorithm, the null model is a substitution matrix that is estimated using phyloFit on the whole genome. The alternative model is based on the estimated substitution matrix plus a newly introduced scaling parameter *ρ* that is applied to the branch length, such that scaling applies to all rates in the substitution matrix at the same time. In order to apply the scaling to only part of the matrix that corresponds to the insertion and deletion rates (*λ* and *μ*), we did not use PHAST’s original scaling algorithm, and instead optimized the scaling parameter by multiplying it directly to the parameters *λ* and *μ*, before they are plugged into the Eqs. (6), (7), and (9) of Rivas and Eddy (2008). Also, we clarify that for the per site column-likelihood, we only need to include the term from the prior ancestral sequence length distribution: Eq. (25) from Rivas and Eddy (2008). We do not need to include the extra normalization terms collected in the “extra column” (*), nor do we need to marginalize the contributions of possible ancestral residues that have left no trace in extant sequences. Thus, those parts were included in the implementation of phyloFit (likelihood of the whole-alignment dataset), but not included in the likelihood calculation inside phyloP (likelihood of each column in the alignment).

The likelihood ratio test checks whether the model that modifies the indel rates by the scaling parameter *ρ*_indel_ fits the alignment column better than the null model estimated from the whole data. *p*-values for the likelihood ratio tests are calculated assuming *χ* ^2^ distribution of the LRT test statistic. If the model with *ρ*_indel_ fits the column significantly better, we infer that the site is under conservation (*ρ*_indel_ < 1) or acceleration (*ρ*_indel_ > 1) depending on the estimated value of *ρ*_indel_. We also report a score, following the convention of PHAST, which is a log transformation of the *p*-value multiplied by ±1 depending on conservation (+1) versus acceleration (−1). The score from the first model comparison is called “phyloP score” in PHAST. We call the score from the second model comparison “indelP score” (Table 2). By scaling the indel rates directly instead of scaling the branch length, the software allows for the first time to estimate rate accelerations specific to indel mutations, independent of nucleotide substitutions. The software with the extended version of phyloFit and phyloP can be found at https://github.com/HanLabUNLV/Phasterate.

### Influence of the Length of Gaps on the Estimation of Insertion Rates and Deletion Rates

One important factor we need to consider when interpreting the rate of insertions and deletions estimated is the difference in the total length of the two events. There is a limitation in our model in that we consider each site as independent events when estimating the indels. So, a deletion of length *n* is considered as *n* independent events, instead of one event that spans *n* sites. This leads to overestimation of the rates, and rate estimation is strongly influenced by the total length of insertions or deletions in the dataset. This limitation is inherent to any indel model that models indels as independent events on a per-residue basis. The consequences of this limitation can be seen in our results, where we compare the estimated rates across three different datasets that are filtered by different lengths of contiguous gaps in the alignment.

In order to examine the effect of the length of indels, we estimated the total length of insertions versus deletions in our data. We used the mostly likely states at the inner nodes to infer insertions or deletions along the trees of all the gene families. The most likely states are inferred based on their likelihood value while estimating the global insertion and deletions rates on the total dataset. Based on implicit parsimony, if the parent node has the highest likelihood value for a gap, while the child node has the highest likelihood value for a residue, we count it as an insertion event, while the opposite pattern is counted as a deletion event. These events are counted along the branches across the total alignment. This allowed us to count the total length of the deletions versus insertions in the dataset.

Based on the model *F84ε*-*relaxed*, the total length of deletions is six to seven times greater than insertions in the data; thus, overestimation impacts deletions more severely. We can see this effect in the estimated substitution matrix where we observe an order of magnitude larger rate of deletions compared to rate of insertions. This difference can be partially explained by this limitation of the model.

This limitation and the resulting overestimation also impact our estimation of the scaling parameter. The rates are estimated at the level of the whole dataset, but the scaling is applied at the level of each residue. In order to weight the scaling per residue appropriately, we decided to divide the scaling parameter by the total length of insertions when applying it to the insertion rate, and divide it by the total length of deletions when applying it to the deletion rate. In practice, instead of dividing each by their total lengths, we multiply the scaling of insertion events by the ratio of deletion to insertions for ease of computation.

### Data

To apply the software to sequence data, we compiled cDNA alignments and trees for the ten primate species *Homo Sapiens*, *Pan troglodytes*, *Gorilla gorilla*, *Pongo abelii*, *Nomascus leucogenys*, *Macaca mulatta*, *Callithrix jacchus*, *Tarsius syrichta*, *Microcebus murinus*, and *Otolemur garnettii* from Ensembl version 75 (GRCh37.p13). We retrieved 21,124 gene trees in newick format and 21,124 cDNA alignments corresponding to those gene trees in fasta format. We only retained alignments that have a *Homo Sapiens* sequence and have at least five sequences. Because we are interested in short indel events and not long composite insertion or deletion of whole domains, we cleaned the alignment with GBLOCK (Talavera and Castresana 2007) and filtered the alignment to exclude long contiguous gaps. To assess the effect of the filtering based on length of gaps on our estimation, we used three different filtering criteria, allowing gaps that are shorter than 15, shorter than 30, and shorter than 45 consecutive sites. The resulting dataset has 838 alignments in well-conserved regions with occasional indels shorter than 15 bases, 1162 alignments with indels shorter than 30 bases, and 1419 alignments with indels shorter than 45 bases. The main results of the paper are from the dataset of 1162 alignments with indels shorter than 30 bases, which amounts to a total of 942,411 sites. The alignments, trees, and the results including the calculated scores can be found at https://github.com/HanLabUNLV/PhasterateData.

## Results

### The Gap-Extended HKY Model Overestimates Insertions Compared to Deletions

Before implementing the *F84ε* model, we tried the default gap-extended model (HKY + G) within PHAST to see if we could apply the scaling parameter (*ρ*_indel_) to HKY + G and get appropriate results. Below is the substitution matrix for HKY + G, and the model with the scaling parameter multiplies the rates in the ‘–’ row and ‘–’ column with *ρ*_indel_
4


HKY + G substitution rate matrix estimated for the primate alignments is as follows:5


Unfortunately, we found that HKY + G, which considers gap as the fifth character and follows the HKY model, did not behave as we expected. The estimation of the substitution matrix was strongly influenced by the observed distributions of residues and gaps, *π* (A, C, G, T, –) = (0.2522, 0.2578, 0.2609, 0.2191, 0.0099), which are in turn used as prior distribution in the likelihood calculation. Because gaps are rarer than residues in our dataset, the rate of gap turning into a residue ($\sigma \pi_{ACGT}$: insertion event) was estimated to be two orders of magnitude higher than the rate of residues turning into a gap ($\sigma \pi_{ - }$: deletion event). The total length of insertions was inferred to be 38,931 bases and the total length of deletions was 14,647 bases based on implicit parsimony. This pattern of greater total insertion length than total deletion length is opposite of the results from model *F84ε* or *F84ε*-*relaxed,* and is unexpected based on observations of deletion bias in several genomes (de Jong and Ryden 1981; Gu and Li 1995; Tao et al. 2007)

### The Generative Model of F84*ε* Overestimates Deletions Compared to Insertions When Gaps are Allowed to be Clumped in the Alignments

Observing the misleading results from HKY + G, we decided to use the model *F84ε* of Rivas and Eddy (2008) as an alternative. Below is the description of the substitution matrix from Rivas and Eddy, where *R* follows the matrix of *F84*, and *δ*_*ij*_ is valued one if *i* = *j* and zero otherwise.6


The parameters estimated based on the primate alignments are *λ* = 0.006871; *μ* = 0.054412; *α* = 0.512803; and *β* = 0.487197, with a prior residue distribution *π* (A, C, G, T) = (0.254698, 0.260464, 0.263575, 0.221263) and a parameter for the geometric distribution of ancestral sequences *p* = 0.998058. These parameters correspond to the substitution matrix as follows:7


The model *F84ε* did not suffer from the problem of large influence of prior distributions on the column-likelihood that we experienced with HKY + G. The main reason is that the probability of a site under *F84ε* only relies on the prior distribution of residues (non-gaps) and does not take into account the prior distribution of gaps into the equation [see Eq. (25) of Rivas and Eddy (2008)]. The length of the sequence is instead modeled with the parameter of the geometric distribution of ancestral sequences *p*. But, *F84ε*, as designed, had an assumption that no more than one insertion event may occur in any given column, which was inappropriate for our purpose. This assumption is appropriate when the alignment splits non-homologous insertions into different sites, as is done by alignment programs like PRANK (Löytynoja and Goldman 2005). But, in our problem, we intend to clump independent indel events into the same sites in the alignment, in order to estimate accelerated rates of indel events on a per column basis. When there is a column that can be explained with more than one independent insertion events, model *F84ε* was required to infer even more counts of deletion events on alternative branches to avoid inferring more than one insertion (see Supplementary Fig. 1a). This resulted in an overestimation of deletions, resulting in insertions with a total length of 8981 bases and deletions with a total length of 72,874 bases based on implicit parsimony. Thus, we modified this model to relax the assumption, and allow multiple insertion events in one column. We call this modified version of *F84ε*, *F84ε*-*relaxed*.

### F84ε-Relaxed Estimated Slightly Higher Rate of Insertions Compared to F84ε, but Inferred the Smallest Number of Total Events Measured by Length

The parameters estimated based on the primate alignments are *λ* = 0.008509; *μ* = 0.061660; *α* = 0.513467; *β* = 0.486533, with a prior residue distribution *π* (A, C, G, T) = (0.254698, 0.260464, 0.263575, 0.221263) and a parameter for the geometric distribution of ancestral sequences *p* = 0.998058. These parameters correspond to the substitution matrix as follows:8


The difference in the parameter estimates is minor. But, the difference in the model assumption, and the corresponding changes in the formulas (1), (2) and (3), leads to a large difference in the total length of inferred insertions and deletions, because now the model does not have to infer multiple deletions instead of insertions (compare Supplementary Fig. 1a, b). Total length of insertions was inferred to be 4506, and the total length of deletions was inferred to be 26,577, inferring smaller number of events than either of the models we explored above based on the same dataset.

### Comparing *F84ε*-*relaxed* Versus *F84ε*-*relaxed* + *ρ*_indel_, We Identified Regions That Have Significantly Higher Indel Rates Independent of Nucleotide Substitution Rates

We estimated the extended *F84ε*-*relaxed* with the scaling parameter *ρ*_indel_ across 1162 alignments containing 942,411 sites. Likelihood ratio test on each site comparing the models *F84ε*-*relaxed* versus *F84ε*-*relaxed* + *ρ*_indel_ identified around 2 % of sites that are under indel rates significantly different from the null model with a significance level of *α* = 0.05. Using a Bonferroni-corrected significance level of *α* = 5.3e−8, there were 102 sites that had significantly different indel rates (Table 3).

**Table 3:** Number of sites with significantly different indel rates and nucleotide substitution rates

Type of event	*α*	Significant sites	Total sites
Indel	0.05	19,134	942,411
Indel	5.3e−8	102	942,411
Nucleotide substitution	0.05	47,243	942,411
Nucleotide substitution	5.3e−8	177	942,411

Number of sites with a significant likelihood ratio test with and without correction for multiple testing

**Fig. 1:**
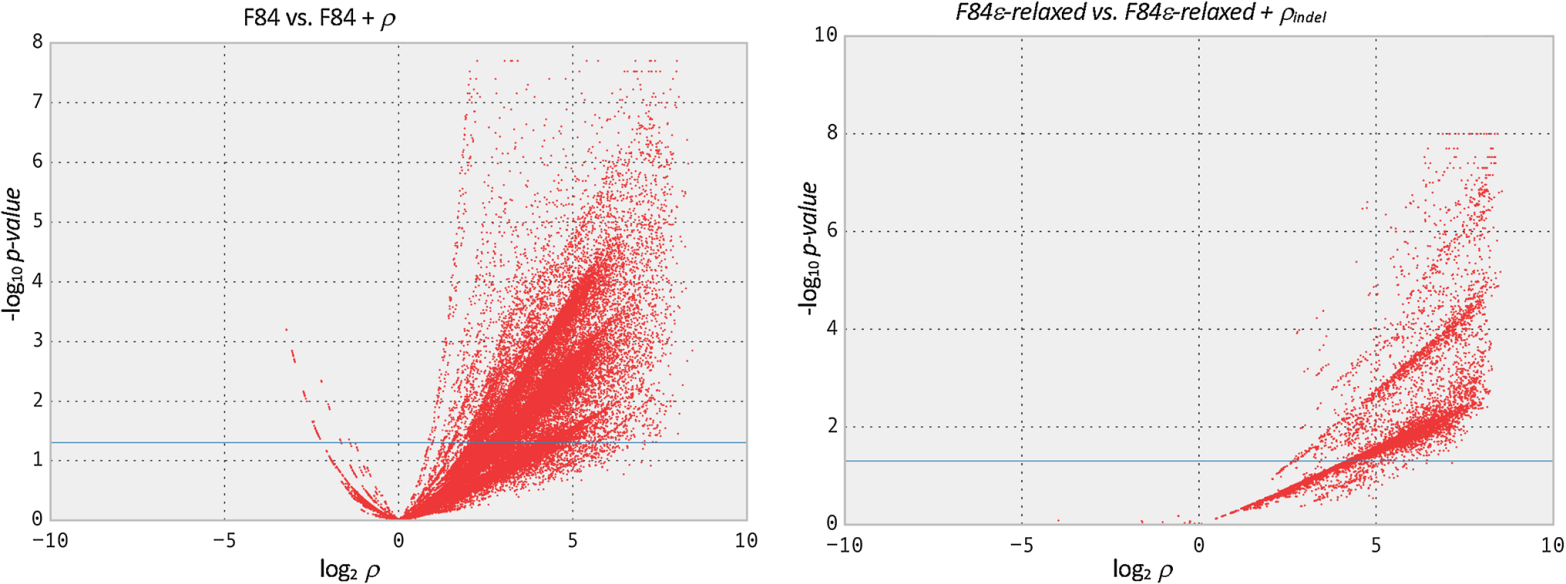
Volcano plot of the likelihood ratio test and the estimated scale parameter for each site in the alignments. Plot of significance versus scaling resulting from the model comparison on 942,411 sites. **a** *F84* versus *F84* + *ρ* tests for significantly different rates of nucleotide substitutions. **b** *F84ε*-*relaxed* versus *F84ε*-*relaxed* + *ρ*_indel_ tests for significantly different rates of indels. Positive values in the *X*-axis represent sites with accelerated rates (scaling > 1), while negative values in the *X*-axis represent sites with conserved rates (scaling < 1). *Y*-axis represents the *p*-value from the likelihood ratio test of the model comparison

The sites that have significantly different rates were all found to have accelerated rates and not conserved rates as shown in the volcano plot in Fig. 1. There are sites with small *p*-values that have scaling parameter *ρ*_indel_ > 1 (accelerated rates), but sites with small *p*-values with scaling parameter *ρ*_indel_ < 1 (conserved rates) are non-existent. This is because for the dataset we constructed, a majority of the columns in the alignment were columns full of residues and no gaps, so the null model estimated on the whole dataset was mostly representing sites with no indel events. Since columns full of residues with no gaps are as conserved as sites can be for indel events, we could not identify any sites that are more conserved than the null.

**Fig. 2:**
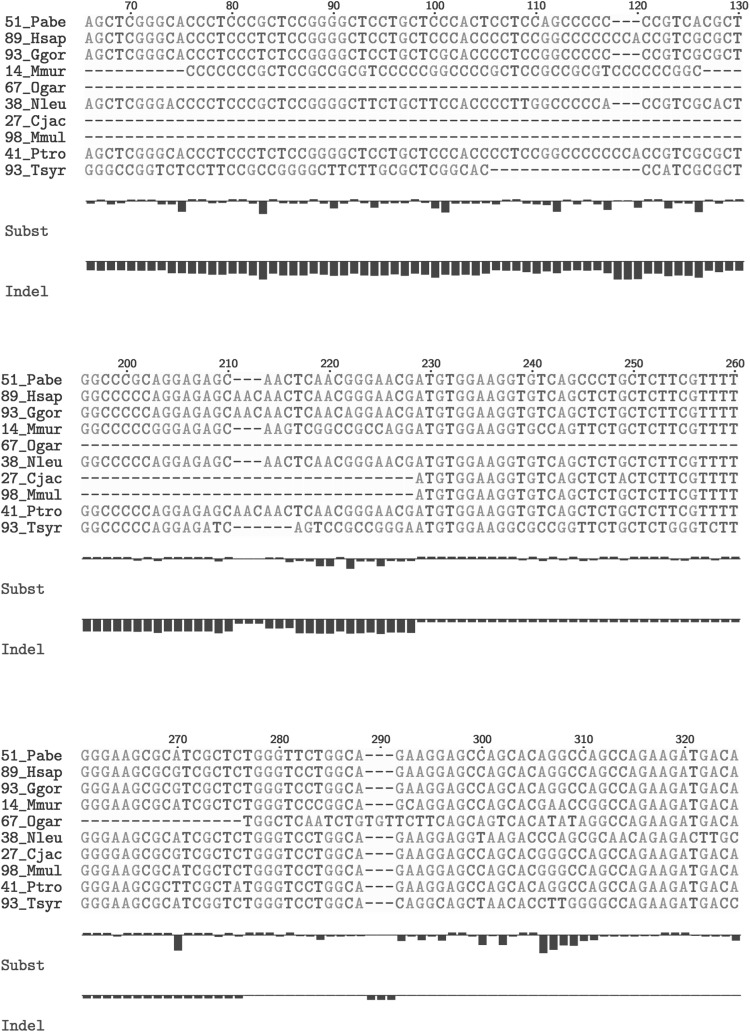
phyloP score and indelP score for gene family podoplanin. Alignment of an example gene family podoplanin (PDPN) with phyloP scores and indelP scores for each site. Scores are calculated by log(*p*-value) multiplied by +1 (conservation, scaling < 1) or −1 (acceleration, scaling > 1). Reference line represents scores that are zero (*p*-value = 1), while the scales of the bars are drawn by normalizing the scores for each family in the range of [− 50, 50]

We visualize the indelP score and the phyloP score for an example gene family of podoplanin (PDPN) to show how the scores are distributed across the alignment as shown in Fig. 2. The scales of the bars are drawn by normalizing the scores for each family in the range of [− 50, 50], so that the bars are only relative to each other within the family, and not across families. Columns 118–120 and columns 211–213 both have same numbers of species with residues versus gaps. Yet, columns 118–120 show accelerated indel rates, while columns 211–213 show indel rates not very different from the null estimate. The different phylogenetic distributions of the residues versus gaps in the two regions lead to the difference in indel rate estimation.

**Fig. 3:**
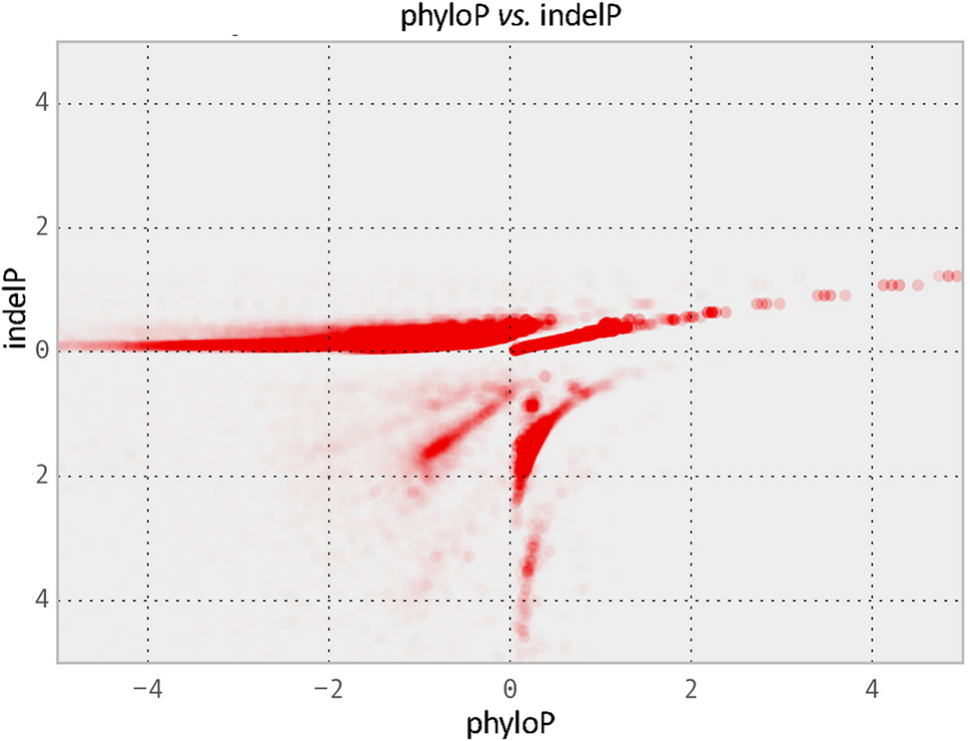
Relationship between phyloP score and indelP score. Plot of phyloP versus indelP scores for 942,411 sites

Those sites that were identified to have significantly different indel rates did not overlap with the sites that had significantly different nucleotide substitution rates (Table 4), confirming that we can identify indel rate acceleration independent of nucleotide substitution rate acceleration. Only 8 % of the sites with significantly different indel rates also experienced significantly different nucleotide substitution rates. Another way to look at the independence between the measure of nucleotide substitution rate change versus indel rate change is to plot the phyloP score against the indelP Score. The relationship between nucleotide substitution rates and indel rates is correlated for a subset of sites, but shows deviation in other sites (Fig. 3).

**Table 4:** Overlap between sites with significantly different indel rates and sites with significantly different nucleotide substitution rates

*α*	Significant for indels and nucleotide substitutions	Significant for indels, but not significant for nucleotide substitutions	Not significant for indels, but significant for nucleotide substitutions	Not significant for indels nor for nucleotide substitutions
0.05	1509	17,544	45,653	877,624
5.3e−8	0	102	177	942,132

Number of sites with significantly different rates for indels and nucleotide substitutions at significance level of 0.05 and 5.3e−8 (Bonferroni-corrected)

### Effect of Gap Filtering on the Estimation of Parameters

To understand the effect of the amount of gaps in the data on the estimated parameters, we ran the software with three different alignment filtering criteria: with at most 15 contiguous gaps, at most 30, and at most 45 contiguous gaps. In Table 5, we can see how the alignments are cleaned and filtered affects the rate estimation, as the lengths of gaps are increased in the alignments, the estimated rates of insertions (*λ*) and deletions (*μ*) are also increased. Rates of nucleotide substitutions (*α* and *β*) are not affected by the amount of gaps, as expected.

**Table 5:** Comparison of results based on three different alignment data

Filtering	*λ*	*μ*	*α*	*β*	*p*	*π* (A, C, G, T)
15 gaps	0.0066	0.0371	0.5449	0.4551	0.9980	0.25, 0.26, 0.26, 0.22
30 gaps	0.0085	0.0617	0.5135	0.4865	0.9981	0.25, 0.26, 0.26, 0.22
45 gaps	0.0090	0.0947	0.5018	0.4982	0.9981	0.25, 0.26, 0.26, 0.22

Filtering	Total alignments	Total sites	Significant sites for indels (α = 0.05)	Bonferroni-corrected significant sites	Total insertions inferred	Total deletions inferred
15 gaps	838	647,478	9126	110 (*α* = 7.7e−8)	2195	8920
30 gaps	1162	942,411	19,134	102 (*α* = 5.3e−8)	4506	26,577
45 gaps	1419	1,236,027	25,969	583 (*α* = 4.0e−8)	7275	62,210

Estimated parameters and results of the indel model comparison for alignment data filtered by different amount of gaps. Estimation for rate of insertions (*λ*) and deletions (*μ*) is influenced by the amount of gaps in the dataset. More gaps in the data lead to higher rates of insertions and deletions, and larger number of total insertions and deletions in length

### Misaligned Regions Containing Gaps can Lead to Erroneous Identification of Sites with Significantly Accelerated Indel Rates

**Fig. 4:**
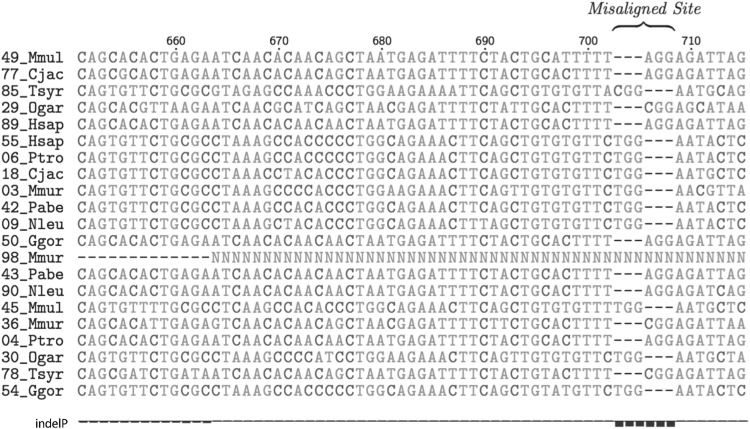
Example of misalignment leading to significant indelP scores. Misalignment in the sequence data can look like accelerated indel rates

As is the case with any sequence models, our method relies on accuracy in sequence alignment. But, since we are trying to estimate accelerated indel rates, and regions containing gaps are especially prone to alignment errors, the negative effect of misalignment is more severe for our problem. Currently, we do not have a solution to automatically take care of this problem. We suggest that the user has to manually check for any sites that are identified to be accelerated in indel rates to make sure the regions are not misaligned. Figure 4 is an example of a possible misalignment that shows significant indelP Scores.

## Discussion

Here, we developed a new software that can estimate accelerated rates of indels independent of rates of nucleotide substitution. Before, evolutionary constraint in molecular evolution was mainly studied in context of nucleotide substitutions. But evolutionary constraint for indel mutations is not necessarily equal to constraint for nucleotide substitutions. Using the software, we can disentangle the two concepts and measure the rates separately. We can estimate the global rate of insertion and deletions for multiple sets of alignments, and afterwards identify sites that have significantly higher rates of insertions and deletions compared to the global rate estimated. Conservation metrics such as phyloP scores have proven to be useful in identifying functional elements in the genome, and predicting deleterious effects of mutations. Whether indel-specific rate metrics can be utilized in a similar way will need to be explored in the future. One difference with indels compared to nucleotide substitutions is that while it is relatively easy to identify regions accelerated for indels, practically it is difficult to identify regions conserved for indels as shown with our results. This may limit the utility of the metric in predicting the deleterious effect of indel mutations in the region. Another difference between indels and nucleotide substitutions is that indels have larger variance in their mutation rate across the genome, such that regions with accelerated rate of indels may be difficult to interpret. Regardless, the ability to measure accelerated indel rates independent of nucleotide substitution rates will contribute to our understanding of how proteins evolve through indel mutations.

## Availability

The software can be found at https://github.com/HanLabUNLV/Phasterate. The data associated with the study can be found at https://github.com/HanLabUNLV/PhasterateData.

## Electronic supplementary material

Below is the link to the electronic supplementary material.

Supplementary material 1 (PDF 493 kb)
