# Supplementary material for: Measuring Accelerated Rates of Insertions and Deletions Independent of Rates of Nucleotide Substitution
Source: J Mol Evol. 2016 Oct 21;83(3):137–46. doi: 10.1007/s00239-016-9761-9 (PMC5080320; doi:10.1007/s00239-016-9761-9)
Supplement: Supplementary file 1 — Supplementary material 1 (PDF 493 kb) [file 239_2016_9761_MOESM1_ESM.pdf]

Supplementary Materials

An artificial alignment and an artificial tree has been created to show the effect of the assumption in *F84ε* that allows at most a single insertion per site. The artificial tree is built to be representative of our data by choosing, for each branch of the primate phylogeny, the mean of the corresponding branch length across all trees in our dataset. The following newick format represents the tree used: (((((((Hsap:0.002886, Ptro:0.002769):0.0010185, Ggor:0.0039045):0.0048765, Pabe:0.008781):0.001323, Nleu:0.010104):0.007838, Mmul:0.017942):0.0099, Cjac:0.033058):0.028135, Tsyr:0.061818):0.014038,(Mmur:0.06649, Ogar:0.095559):0.025367);

The artificial alignment is a column representing two independent insertion events, one in the marmoset branch, and one in the human branch. For this artificial column, we show the calculated probability for each state in the inner nodes of this phylogeny based on the substitution matrix estimated for *F84ε* (Supplementary Figure 1. a) and for *F84ε-relaxed* (Supplementary Figure 1. b). These substitution matrices are estimated from our whole dataset comprising of 1,162 primate alignments and phylogenies.

We can see that under *F84ε* the column with two independent insertions has an extremely small probability close to zero. The ancestral nodes of gorilla to macaque has gaps as the most likely state, but once a non-gap residue is observed at the marmoset leaf, the probability of the states at the ancestor of marmoset becomes close to zero. The ancestral node of marmoset is unlikely to be an 'A' because based on the indel rate estimated from the whole dataset, it is unlikely that the 'A' to '–' transition will happen during the short branch leading to the ancestral node of macaque. The node is unlikely to be a '–' either because if the node is a gap, there will be more than one insertion events leading to marmoset and human which is not allowed. Thus the column likelihood becomes extremely small.

When we introduce the indel scaling parameter to *F84ε*, the ancestral node of marmoset now has a higher probability of being an 'A' residue since the rate of 'A' to '–' transition has increased due to the scaling, but the node still has a very low probability of being a '–' because the model does not allow more than one insertion per column. Because the null model had an extremely low probability, introducing the scaling increases the likelihood significantly, and the scaling parameter is estimated to be very large (ρ_{indel} = 435.178). Having 'A' as the most likely state at the ancestral nodes of marmoset means that multiple 'A' to '–' deletions are inferred at the branches leading to tarsier, mouselemur, bushbaby as well as the ancestral node of macaque, instead of two insertion events.

This effect is not observed under *F84ε-relaxed* after we relax the assumption of *F84ε* by changing the formulas (1)(2) and (3). Now ancestral node of marmoset has a non-zero probability of being a '–', and as we move up the tree and observe more '–' in the leaves of tarsier, mouse lemur, and bushbaby, '–' becomes the most likely state in the inner nodes, unlike what is observed under *F84ε*. Inferring '–'s at the ancestral nodes of tarsier, and at the

ancestral node of macaque means more than one insertion is inferred in this column. When we introduce the scaling parameter, the likelihood increases, but not as much as in *F84ε*, and the scaling parameter is estimated to be smaller than in *F84ε* (ρ_{indel} = 181.245). Because '–'s are the most likely states at the nodes ancestral to marmoset, it is not necessary to infer multiple deletions.

Supplementary Figure 1 Example phylogeny and alignment showing the effect of the assumptions in *F84ε* that allows at most a single insertion per site.

The probability of each state (A,C,T,G,–) is shown for each node in the phylogeny. Top lines at the inner nodes show the probabilities under the null model and bottom lines show the probabilities under the alternative model including the scaling parameter. a) under models *F84ε* and *F84ε+* ρ_{indel}. b) under models *F84ε-relaxed* and *F84ε-relaxed* +ρ_{indel}

a)

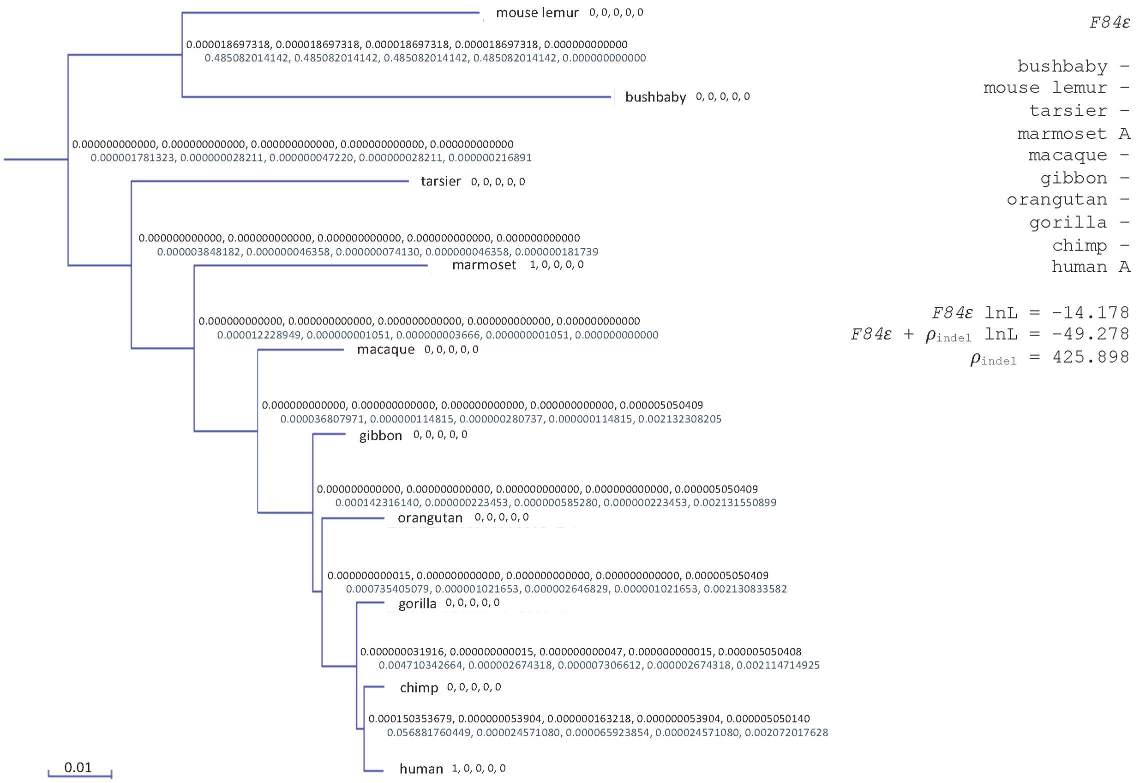

b)

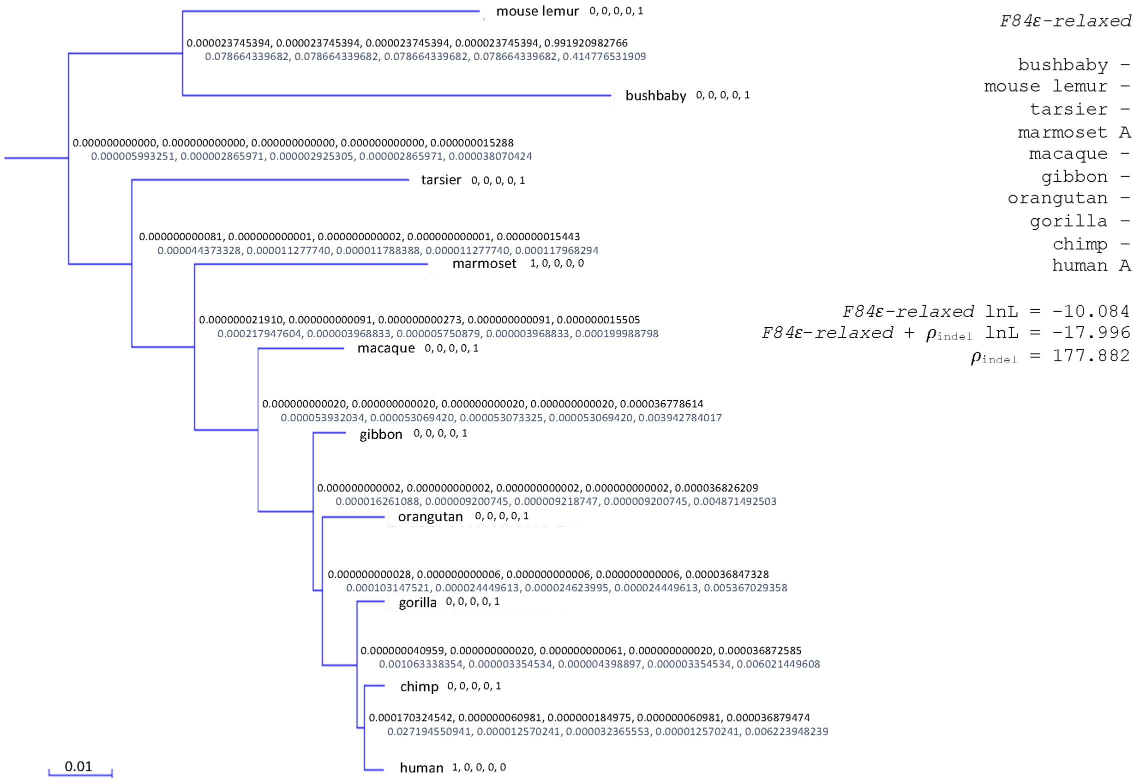

REFERENCES

Rivas E, Eddy SR. 2008. Probabilistic Phylogenetic Inference with Insertions and Deletions. PLoS Comput. Biol. 4:e1000172.
